# Low Doses of *Cuscuta reflexa* Extract Act as Natural Biostimulants to Improve the Germination Vigor, Growth, and Grain Yield of Wheat Grown under Water Stress: Photosynthetic Pigments, Antioxidative Defense Mechanisms, and Nutrient Acquisition

**DOI:** 10.3390/biom10091212

**Published:** 2020-08-20

**Authors:** Qasim Ali, Rashida Perveen, Mohamed A. El-Esawi, Shafaqat Ali, Syed Makhdoom Hussain, Maira Amber, Naeem Iqbal, Muhammad Rizwan, Mohammed Nasser Alyemeni, Hamed A. El-Serehy, Fahad A. Al-Misned, Parvaiz Ahmad

**Affiliations:** 1 Department of Botany, Government College University Faisalabad, Faisalabad 38000, Pakistan; mairamber65@yahoo.com (M.A.); drnaeem@gcuf.edu.pk (N.I.); 2 Department of Physics, University of Agriculture Faisalabad, Faisalabad 38000, Pakistan; 2007ag942@uaf.edu.pk; 3 Botany Department, Faculty of Science, Tanta University, Tanta 31527, Egypt; mohamed.elesawi@science.tanta.edu.eg; 4 Department of Environmental Sciences and Engineering, Government College University, Allama Iqbal Road, Faisalabad 38000, Pakistan; mrazi1532@yahoo.com; 5 Department of Biological Sciences and Technology, China Medical University, Taichung 40402, Taiwan; 6 Department of Zoology, Government College University Faisalabad, Faisalabad 38000, Pakistan; drmakhdoom90@gmail.com; 7 Botany and Microbiology Department, College of Science, King Saud University, Riyadh l1451, Saudi Arabia; mnyemeni@ksu.edu.sa (M.N.A.); parvaizbot@yahoo.com (P.A.); 8 Department of Zoology, College of Science, King Saud University, Riyadh l1451, Saudi Arabia; helserehy@ksu.edu.sa (H.A.E.-S.); almisned@ksu.edu.sa (F.A.A.-M.); 9 Department of Botany, S.P. College, Maulana Azad Road, Srinagar, Jammu and Kashmir, Pune 190001, India

**Keywords:** antioxidant enzymes, biostimulant, lipid peroxidation, grain yield, seed germination

## Abstract

The present study was conducted to investigate the effects of *Cuscuta reflexa* extract (CRE) on the activities of germination enzymes, seed germination vigor, biomass production, physio-biochemical attributes, and seed yield of water-stressed wheat plants. Different levels of CRE (0, 10, 20, 30, 40, and 50%), including water soaking, were used as seed priming. Water stress negatively affected the seed germination, germination enzyme activities, growth, yield, and different physio-biochemical attributes of wheat plants. Low doses of CRE (10, 20, and 30%) ameliorated the adverse effects of water stress on seed germination attributes, and activities of germination enzymes, but negative impacts were recorded at higher doses (40 and 50%) of CRE. Water-stressed wheat plants grown from seeds pre-treated with low doses of CRE also showed better growth and yield as compared with non-treated ones, and that was associated with an improvement in water relations, photosynthetic pigments, nutrient acquisition, reduced lipid peroxidation, and better antioxidative defense mechanisms. The maximum increase in seed yield was 14.77 and 12.32%, found in plants grown from seeds treated with 20% and 10% CRE, respectively. In conclusion, it is suggested that using low doses of CRE as seed priming can contribute to better wheat yield under water stress, especially in semi-arid and arid areas.

## 1. Introduction

In the present scenario, changing environmental conditions at a global level, along with deficiency of fresh water for irrigation, represent a major limiting abiotic factor for meeting the world food demand. This issue threatens the world crop production through multiple ways. Water stress affects each stage of plant life, starting from germination to grain filling [1,2]. Seed germination and seedling vigor are of prime importance [3] because they are necessary for better and uniform crop stand establishment [4], resulting in better final production. Under soil water deficit, they are seriously disturbed, leading to severe crop yield losses worldwide [5]. Water stress severely disturbs the plant water relations, including seed germination. It also disturbs leaf photosynthetic processes through stomatal or non-stomatal factors, which cause the oxidative stress as a resulting reduction in growth and yield [6,7]. 

Among agronomic crops, wheat is being used as a staple food in most parts of the world and is considered the most important one for meeting the world food demand [8]. However, its production faces the water shortage issue at different growth stages, especially in the rainfed conditions as well as areas with limited irrigation. Dry environmental conditions in rainfed areas have further threatened wheat production. To overcome the issues of poor germination and emergence for better crop production under prevailing soil water deficit environment, different strategies are being employed, including the selection of crop varieties with inherent potential of better germination under limited water supply along with better agronomic practices [9]. However, regarding the selection of genotypes for better yield, most high yielding wheat crop varieties do not perform well under water stress [10]. Among others, seed priming through different ways is being employed excessively for better crop stand establishment [4]. These priming techniques include hydro-priming, chemo-priming, and halo-priming. Seed priming has gained a great interest due to its better economic outcomes. However, most of the seed priming techniques include the use of different chemicals that are not eco-friendly. Therefore, the current trend is to increase the use of organic and eco-friendly compounds as seed priming agents, which have an effective role in plant stress tolerance [11,12]. These include the use of amino acids [13], vitamins [14,15], and sugars [6], individually or in combination with plant breeding-based molecular genetic techniques [16,17], for effectively combating the adverse effects of stresses in plants. So far, various studies recommended the use of above-mentioned organic compounds for combating stress-inducible adverse effects on crop plants for better production [15,18,19]. However, all of these organic compounds are not cost effective nor easily available to the farmers. Recently, in parallel with the exogenous use of eco-friendly synthetic organic compounds for the induction of stress tolerance in crops, plant-based extracts have been used as a rich source of organic compounds, as reported in details in the special issue of Colla and Rouphael [12] and termed them as biostimulants. Recently the term “botanicals” was coined for the plant-based biostimulants by du Jardin [20]. Any extract or microorganism included in biostimulants category is being used in plants for inducing stress tolerance, enhancing crop quality traits, and boosting the plant nutritional efficiency without considering its nutrient composition. It has been estimated that market demand of biostimulants will increase to 3 billion, until 2020 [21]. In some earlier studies, it has been reported that the exogenous use of various plant extracts through different ways found effective in improving the stress tolerance of crop plants [11,12,20,22]. For example, it has been found by Habib et al. [23] and Noman et al. [11] that sugar beet root extract is helpful for the induction of salt and drought tolerance in okra and wheat, respectively. Additionally, Narayanan et al. [24] reported that the use of *Prosopis* leaf extract as pre-sowing treatment improved the seed yield and quality of sesame. Latif and Mohamed [25] also reported the effective role of moringa leaf extract as a foliar spray in enhancing antioxidative defense mechanisms and yield components of salt-stressed common bean plants. Yasmeen et al. [26] also reported that foliary-applied moringa leaf extract significantly improved the grain yield of salt-stressed wheat plants. Furthermore, the combined application of *Brassica* and moringa leaf extracts positively influenced the yield components of canola [27]. The extracts of some other plant species have been also used as exogenous bio-stimulants for better crop production. However, all plants being used as biostimulants are not economically important ones. Nevertheless, a wide range of biostimulants has also been reported to be beneficial for better plant growth, not only under normal growth conditions, but also under stressful environments [20,28,29]. Furthermore, in earlier studies, it has been found that the induction of stress tolerance was biostimulants dose-dependent, and mostly these studies are mainly focusing on the plant growth increments in parallel with the modulation of physio-biochemical attributes.

In view of literature review, it has been hypothesized that seed priming with *Cuscuta reflexa* extract (CRE) may be helpful in improving seed germination, seedling emergence, and vigor that might improve the grain yield of wheat under water deficit conditions. *Cuscuta reflexa* is a perennial parasitic and epiphytic herb belonging to the family Convolvulaceae. It is also used as a medicinal herb in some Asian countries. It is rich in ascorbic acid (AsA), tocopherols, phenolics, flavonoids, amino acids, and mineral nutrients [30,31]. Until now, very little, or no attention, was given to the beneficial uses *Cuscuta reflexa*. Hence, the present study was conducted with the aim to assess at what extent seed priming with CRE will be helpful to modulate the adverse effects of water stress on wheat seed germination, seedling emergence, biomass production, and seed yield. Furthermore, the plant responses in terms of the modulations of physio-biochemical attributes, antioxidative defense mechanism, and nutrient uptake were also studied.

## 2. Material and Methods

### 2.1. Preparation of CRE Extract

For the preparation of plant extract, fresh plants of *Cuscuta reflexa* growing parasitically on *Ziziphus mauritiana* plants were collected. Before taking the extract, the *Cuscuta reflexa* plants (3 Kg fresh biomass) were washed well and cut into small pieces. Then, the extract was obtained using an electric blender to crush the plant followed by screw pressing. The extract was then filtered using cheesecloth. The different dilutions/concentrations (0, 10, 20, 30, 40, and 50%) of the extract were made with dH_2_O (v/v) and used as seed priming. For making 10% concentration, 10 mL of filtered CRE was mixed with 90 mL dH_2_O, and so on, for the other CRE concentrations. The remaining extract was stored at −10 °C for different biochemical analysis as presented in Table 1.

### 2.2. Composition of Extract

#### 2.2.1. Estimation of Total Phenolic Content (TPC) in CRE

For the estimation of TPC in CRE, the method ascribed by Singleton and Rossi [32] was followed using the Folin–Ciocalteu phenol reagent with some modifications. The filtered CRE was mixed well with acetone (1.5:3.5 v/v, respectively) for 30 min and centrifuged at 10,000× *g*. The supernatant was diluted with dH_2_O to reduce the proportion of acetone to 7%. The diluted extract was then mixed with 2.5 mL of Folin–Ciocalteu phenol reagent, incubated at room temperature for 5 min and mixed with 2 mL of Na_2_CO_3_ (7.5%). The mixture was then heated at 50 °C for 15 min. After cooling in ice bath, the absorbance of the resultant solution was read at 765 nm. For the quantification of TPC, gallic acid was used as a standard with a range of concentrations. The results were expressed as mg GAE/100 g fresh weight.

#### 2.2.2. Estimation of Total Flavonoid Content (TFC) in CRE

Total flavonoids in the CRE were estimated following the method given by Sultana et al. [33] with some modifications. In the present study, the filtered CRE was diluted with absolute methanol (1:4 v/v) and centrifuged at 10,000× *g* for 10 min. The supernatant was then mixed with 0.3 mL of 5% NaNO_2_. After 5 min, the resultant mixture was reacted with 0.3 mL of 10% AlCl_3_. After 6 min, the resulted solution was mixed with 1 M NaOH (2 mL) and 2.8 mL of ddH_2_O. After incubation for 40 min at room temperature, the absorbance of the final colored solution was read at 430 nm. The quantity of the flavonoids was estimated using the range of Catechin standards.

#### 2.2.3. Estimation of Ascorbic Acid (AsA) Content in CRE

The method described by Mukherjee and Choudhuri [34] was used for the estimation of AsA content in CRE with slight modifications. Briefly, 10 mL of CRE was mixed with 50 mL of 5% metaphosphoric acid and acetic acid solution followed by centrifugation at 10,000× *g* for 10 min at room temperature. The supernatant (12 mL) reacted with 2% acidic dinitrophenyl hydrazine (6 mL) solution followed by the addition of one drop of 10% thiourea. The mixture was boiled for 20 min in a water bath followed by the addition of 80% H_2_SO_4_ (5 mL) after cooling at 0 °C. The absorbance of the resulting solution was read at 530 nm. The quantity of ascorbic acid was determined using a standard curve drawn with the series of known concentrations of ascorbic acid.

#### 2.2.4. Estimation of Carotenoids in CRE

Determination of the carotenoids in CRE was done using the acetone-ether extraction method. Briefly, the CRE and the acetone were mixed in equal volumes (1:1). After shaking well, the obtained acetone layer was mixed well with five volumes of petroleum ether (1:5). Then, the petroleum ether layer was separated and the absorbance was read at 450 nm. The following equation was used for the quantification of carotenoids:

*β*-carotene = A × df × V∕E1% 1cm × w

where V is the volume of the sample used; (mL), df is the dilution factor, E1% is the coefficient of absorbency (2592 for petroleum ether) 1 cm, A absorbance.

#### 2.2.5. Estimation of Vitamin E Content in CRE

The method ascribed by Backer et al. [35] was followed for the estimation of vitamin E content in CRE with some modifications. Filtered CRE (30 mL) was mixed with ethanol (3:10). After shaking well, 50 mL *n*-hexane/ethyl acetate (9:1) was added to the mixture in a separating funnel and the organic layer was separated. This process was repeated thrice and the organic layers were combined. The material was filtered and dried using N gas at room temperature. The residue was then dissolved in 30 mL ethanol and used for the estimation of vitamin E. Two mL of the final extract was mixed with 2% ethanolic 2 and 2-dipyridyl (200 μL) in dark and 4 mL dH_2_O was added and mixed well. The absorbance of the resultant material was read at 520 nm. The quantification of vitamin E was done by preparing a standard curve from a series of known concentration using pure analytical grade α-tocopherol. 

#### 2.2.6. DPPH Scavenging Activity of the CRE

The DPPH radical scavenging activity of the CRE was evaluated following Brand-Williams et al. [36] with some modifications. The centrifuged CRE was directly used for the estimation of DPPH activity instead of the crude extract. Briefly, 1 mL of 100 mM sodium acetate buffer (pH 5.5), 0.5 mL of an methanolic solution of DPPH (250 µmol/L), 1 mL of absolute methanol, and 50 µL of the CRE extract were mixed and kept in the dark for 30 min at room temperature. The absorbance of the resulted solution was read using a UV-VIS spectrophotometer at 517 nm. The Trolox standard curve was used for the quantification of antioxidant activity.

#### 2.2.7. Ferric Reducing Antioxidant Power (FRAP) of the CRE

The method described by Benzie and Strain [37] was followed for performing the FRAP assay of the CRE. FRAP reagent was prepared by mixing one part of 2-4-6-tripyridyl-s-triazine (10 mM in 40 mM HCl) with 10 parts of acetate buffer (300 mM, pH 3.6) and one part of iron (III) chloride-hexahydrate (20 mM in ddH_2_O). CRE was diluted 1:10 in doubled distilled H_2_O. Then, 300 mL of freshly prepared FRAP reagent was mixed thoroughly with 10 mL of each of the diluted sample. The absorbance of the resultant solution was measured immediately at 593 nm after incubation for 10 min at 37 °C. FRAP results for each sample were calculated using a dilution series of trolox. As a positive control for each assay, 1 mM ascorbic acid standard was analyzed. 

#### 2.2.8. Estimation of Total Soluble Sugar (TSS) Content of CRE

The separation of TSS in CRE was employed following the method described by Homme et al. [38]. The anthrone reagent method was employed for the estimation of TSS content in CRE. The absorbance of the resulting solution was read at 625 nm. The final quantification of TSS was made using a standard curve prepared from the pure standards (200–1000 ppm).

#### 2.2.9. Estimation of Oil Content of CRE

For the estimation of oil content in CRE, the method described by James [39] was followed. Briefly, a known volume of CRE was dried and the oil content was extracted with *n*-hexane as a solvent using the Soxhlet apparatus. After recovering the solvent by rotary evaporator, the flask containing the oil content was oven-dried at 60 °C for 3 min (i.e., to remove residual solvent). The oil content was measured using the equation:

$$\%\;\text{oil}=\frac{W2-W3}{\text{Wt of sample}}\times 100$$

where W2 = weight of flask with extracted oil and W3 = weight of empty flask.

#### 2.2.10. Estimation of Protein Content in CRE

The method ascribed by Ogan et al. [40] was followed for the estimation of proteins in CRE with slight modifications. Briefly, the fresh extract was mixed with 50 mM sodium phosphate buffer (1:1 v/v) having 3 mM sodium metabisulphite (in place of DTT). The mixture was then centrifuged at 5000× *g* for 30 min at 4 °C. The supernatant was then vacuum-filtered using 0.45 µm cellulase acetate HA-membrane. The filtrate was then used for the estimation of proteins following the method of Bradford [41].

#### 2.2.11. Estimation of Fiber Content in CRE

The fiber content in CRE was estimated following the method described by AOAC [42]. To 40 mL of CRE, hot 200 mL 1.25% H_2_SO_4_ was added and boiled for 30 min in a beaker. The solution was then filtered and the residue was washed progressively with boiling water, alcohol, and petroleum ether and transferred to a porcelain crucible. The residue was then dried in an oven at 150 °C to a constant mass. The crucible was then cooled, weighed and put for 2 h at 600 °C in a muffle furnace. After cooling, the crucible was again weighed. The fiber content was estimated using the formula

$$\%\;\text{crude fiber}=\frac{M2-M4}{M2-M1}\times 100$$

where M1 = mass of the empty crucible; M2 = mass of crucible having the sample; M3 = mass of crucible having residue and M4 = mass of crucible having ash after incinerator. 

#### 2.2.12. Determination of Water Content in CRE 

For the estimation of water content in CRE, gravimetric method was employed following AOAC [42]. Hundred mL of pure CRE was taken in an oven-dried pre-weighed conical flask. The flask was then put at 105 °C for 3 h, cooled in a desiccator and weighed. This cooling drying and weighing was repeatedly done until the constant weight was obtained. Then, the equation given below was used to measure the % water content in the CRE:

$$\%\;\text{moisture in CRE}=\frac{W2-W3}{W1}\times 100$$

where, W2 = weight of flask containing sample before drying; W3 = weight of flask after drying of sample and W1 = weight of sample used. 

#### 2.2.13. Determination of Glycine Betaine (GB) Content in CRE 

For the estimation of GB content in the CRE, the method described by Grieve and Grattan [43] was used. One mL of CRE was mixed with 1 mL of 2 *N* HCl and then half mL of this mixture was mixed with 0.2 mL of KI_3_ solution. The mixture was cooled for 90 min after shaking well. Then, 2 mL of ice cooled water and 20 mL of dichloromethane were added in ice-cooled mixture. After shaking well, the supernatant was discarded and absorbance of lower layer was read at 365 nm. The quantification of GB in samples was done by creating a standard curve prepared from pure standards.

#### 2.2.14. Determination of Proline in CRE

The method ascribed by Bates et al. [44] was followed for the estimation of proline in CRE. Briefly, 200 µL of CRE was mixed with 6 M H_2_SO_4_ (2 mL each) and the mixture was reacted with glacial acetic acid and acidic ninhydrin (2mL each) followed by heating at 95 °C in a water bath. After cooling well, 4 mL of toluene was mixed with the reaction mixture and the optical density of colored phase was read at 520 nm. A range of standards (5–25 ppm) was also prepared and run under the same conditions to prepare a standard curve for the estimation of proline concentration in samples. Final proline concentration in samples was quantified using the equation given below:

Proline (µmol g^−1^ Fw) = mL of toluene/115 × µg proline ml^−1^)/sample (g)

#### 2.2.15. Estimation of the Minerals in the CRE

Flame photometer and atomic absorption spectrometry were used for the estimation of minerals in the CRE. Flame photometer (Jenway PFP 7, Essex, UK) was used for the estimation of K, Ca, and Mg contents, while atomic absorption spectrophotometer (Hitachi High Technology Corporation, Tokyo, Japan) was used for the estimation of all other minerals. The content of Cl in the extract was also determined using a Chloride Analyzer (926 Mark 2 Chloride Analyzer Sherwood Scientific Ltd., Cambridge, UK).

### 2.3. Germination Experiment (Study of Seed Germination Attributes and Activities of Germination Enzymes)

The experiment was conducted in a growth room as well as in soil-filled pots under natural environmental conditions. The growth room experiment was conducted using Petri-dishes to find out the effects of seed priming with CRE on seed germination attributes and the activities of different germination enzymes.

The growth room experiment was arranged as two sets of Petri-dishes. One set was specified for the study of seed germination attributes and other one for the activities of germination enzymes. For seed priming, there were five concentrations of CRE applied as 0, 10, 20, 30, 40, and 50%. Seeds of wheat cultivar Galaxy-13 were used in the present study. Seeds were surface sterilized for 10 min with 5% sodium hypochlorite solution before priming with CRE extract. Nine hundred seeds were soaked separately in 200 mL solution corresponding to each level of CRE. Seeds were soaked for 12 h and kept in dark. Seeds were then air-dried for two hours to evaporate the excess moisture. In each group, the Petri-dishes were divided in two sets. One set was allocated as non-stressed and the other one was allocated as stressed one to fulfill the experimental requirements. The Petri-dishes were supplied with the double layer of filter paper. In each group, the set of Petri-dishes specified as non-stressed was supplied with half strength Hoagland’s nutrient solution and the other set of Petri-dishes specified as stressed one was supplied with PEG-8000 solution (16%) prepared in half strength Hoagland’s nutrient solution having osmotic potential −0.66 M.Pa. Each set was comprised a total 18 Petri-dishes with three Petri-dishes specified to each treatment (triplicate) and each group was specified with a total of 36 Petri-dishes. Twenty seeds were sown in each Petri-dish corresponding to the group specified for the study of seed germination attributes, while 80 seeds were sown in each Petri-dish specified for the activities of seed germination enzymes. The whole arrangement of the experiment was done in a growth room at 25 ± 2 °C and the following attributes were studied during the course of experiment by collecting data from the 1st day of the start of experiment. The germination experiment was continued for 7 days until the constant count of the germinating seeds was attained. 

### 2.4. Estimation of Different Germination Attributes

#### 2.4.1. Estimation of Seed Germination Percentage Age (G%)

For the estimation of different germination attributes, the germinated seeds were counted on a daily basis. Seed germination percentage age (G%) was calculated following the formula;

Seed G% = number of germinated seeds/total number of seeds × 100

#### 2.4.2. Estimation of Seed Germination Energy (GE)

For the estimation of seed GE, the method ascribed by Ruan et al. [45] was followed by calculating the % seed germinated on the fourth day relative to total number of germinated seeds sown at the final count in order to calculate the GE.

#### 2.4.3. Estimation of Seed Emergence Index (EI) and Mean Emergence Time (MET)

For the estimation of seed EI, the method given by Association of Official Seed Analysis [46] was followed, while the method ascribed by Ellis and Roberts [47] was followed for the estimation of MET.

#### 2.4.4. Coefficient of Uniformity of Emergence (CUE) and Time to 50% Emergence (E_50_)

For the estimation of CUE, the method described by Bewley and Black [48] was employed while the method ascribed by Coolbear et al. [49] was followed for the estimation of E_50_.

### 2.5. Activities of Germination Enzymes 

From the second set of Petri-plates, fresh seed samples from each Petri-plate were collected with the interval of 24 h, up to 72 h for the assay of the activities of proteases, amylases, and glucosidase.

#### 2.5.1. Activities of Proteases (Pro)

Activities of Pro in germinated seeds were appraised following Ainouz et al. [50]. Ten seeds from each replicate were selected for the extraction of enzymes. The extraction was carried out with cold 1% NaCI solution (prepared in 0.2 mM phosphate buffer having pH 7.0). The germinated seeds were ground well in an ice bath using a pestle and mortar. After centrifugation of the resultant extract at 16,099× *g* for 30 min at 10 °C, one mL of the supernatant was mixed with 5 mL of 1% casein solution prepared in 0.2 M phosphate buffer (pH 6.0) and the resultant solution was incubated at 50 °C for 1 h. The reaction was terminated with the addition of 1 mL of 40% trichloroacetic acid (TCA) solution. The proteolytic activity was found following Lowry et al. [51] by measuring the absorbance at 570 nm.

#### 2.5.2. Activities of Amylases (Amy)

For the estimation of Amy activity, the extraction (ten seedling/replicate) was carried out with cold 1% NaC1 solution prepared in 0.2 mM phosphate buffer (pH 5.5). The obtained supernatant after the centrifugation (11,180× *g* for 10 min) at 10 °C was used for the estimation of the activity of Amy following Chrispeel and Varner [52]. The activity of enzymes was expressed as mg of starch hydrolyzed g^−1^ fresh weight h^−1^.

#### 2.5.3. Activity of Glucosidase (Gluco)

*Enzyme Extraction*

The method described by Peruffo et al. [53] was used for the enzyme extraction. Germinating seeds (in a ratio of 2 mL of buffer/g seed fresh weight) were homogenized in 0.05 M Na-acetate buffer (pH 5.0) prepared with 5 mM ß-mercaptoethanol in an ice bath using the mortar and pestle. After centrifugation at 20,000× *g* at 4 °C, the supernatant was brought up to 80% saturation with NH_4_SO_4_. After stirring well, the solution was centrifuged again at 20,000× *g* at 4 °C. The pellet was re-suspended in 0.05 M Na-acetate solution (pH 5.0), and dialyzed against the same buffer at one tenth of the molarity for 24 h. The dialysate was then concentrated with PEG-40,000 to a final volume of 10 mL.

*Gluco Assay*

The incubation mixture containing 0.1 mL of maltose of desired concentration prepared in McIlvaine buffer having pH 5 following Dawson et al. [54], 0.3 mL of the same buffer, and 0.1 mL of gluco preparation was prepared. The reaction was started by the addition of enzyme. The assay mixture was incubated for 30 min at 37 °C. The gluco activity was determined from the glucose liberated from maltose using the glucose oxidase method [55]. After the addition of glucose reagent, the reaction mixture was incubated for 50 min at 37 °C, and the reaction was terminated by adding 2.5 mL of 5 N HC1 with a vigorous mixing. The absorbance at 525 nm was measured.

### 2.6. Study of Plant Growth, Yield, Physio-Biochemical Attributes, Antioxidative Defense Mechanisms, and Nutrient Acquisition

The second experiment was conducted in soil-filled plastic pots under natural environmental conditions to find out the effects of seed priming with different concentrations of CRE (as used in the germination experiment) on the growth, physio-biochemical and yield attributes of wheat plants grown under water deficit conditions. The experiment followed the Completely Randomized Design (CRD) and each treatment was done in triplicate. The whole experimental setup comprised a total of 36 plastic pots having a depth of 45 cm and a diameter of 30 cm filled with 16 kg sandy loam soil. Before seed sowing, the soil in pots was prepared well by watering and followed by hand digging when the soil was at the field capacity. The CRE-primed seeds were sown when the soil in pots was at 100% field capacity. Fifteen seeds were sown in each pot. After seven days of seed germination, seven plants per pot were maintained by thinning the seedlings. The pots were then divided into two sets (each set having 18 pots and 3 pots per treatment). One set of pots was taken as a non-stressed (normal watering as per the requirement) and the other one was taken as a stressed one supplied with a reduced water to maintain 60% field capacity. In the pots specified for water stress treatment, the soil moisture was maintained on a daily basis at 60% field capacity using the Tensiometer (Irrometer, Model, LT-12 inch, Riverside, CA, USA). Different physico-chemical properties of the soil used in pots were analyzed. The analyzed soil having the saturation percentage of 46%, pH 7.56, P 0.051 mg kg^−1^, K 30 mg kg^−1^, and N 5.9 mg kg^−1^, and EC 0.048 ds/m. After 45 days (vegetative stage before booting stage) of the start of water stress treatment, data for various growth and physio-biochemical attributes were estimated. For the estimation of growth and morphological attributes, two plants from each pot were harvested. After measuring fresh masses of root and shoot, the plants were kept in an electric oven at 70 °C to measure their dry masses as well as for estimation of nutrients content. From the remaining plants, two plants from each pot were harvested and stored at −20 °C for the assessment of different physio-biochemical attributes. For the estimation of yield attributes, the experiment was continued under prevailing water stressed and non-stressed conditions till the maturity of plants.

#### 2.6.1. Estimation of Leaf Chlorophyll (Chl.) and Carotenoid (Car) Contents

For the estimation of leaf Chl. *a*, Chl. *b*, total Chl. and Chl. *a*/*b*, the method described by Arnon [56] was followed. The content of Car was estimated following Kirk and Allen [57]. The extraction of the pigments was done using the 80% acetone. Briefly, fresh leaf material (0.1 g) was chopped and put in 10 mL acetone for overnight at 4 °C and the optical density (OD) of the extract was read at 663, 645 and 480 nm using spectrophotometer (Hitachi U-2001, Tokyo, Japan). The quantities were computed using the specific formulas:

Chl. *a* = [12.7 (OD 663) − 2.69 (OD 645)] × v/1000 × w
Chl. *b* = [22.9 (OD 645) − 4.68 (OD 663)] × v/1000 × w
Total Chl. = [20.2 (OD 645) - 8.02(OD 663)] × v/w × 1/1000
A Car (µg/g FW) = OD 480 + (0.114 × OD 663) − (0.638 × OD 645)
Car = A Car/Em 100% × 100
Emission = Em 100% = 2500
OD = absorbance at respective wavelength
V = volume of the extract (mL)
W = weight of the fresh leaf tissue (g)

#### 2.6.2. Estimation of Leaf Relative Water Content (LRWC)

Fully developed leaf (third from the top) was used for measuring the LRWC. The leaves were excised using scissors and their fresh weight was measured. Leaves were then dipped in distilled water for 6 h after marking a specific number. The leaves were then taken out from water, the excess water on their surface was adsorbed, and their turgid weights were measured. Then, the dry weights of these leaf samples were measured after oven-drying at 70 °C for 48 h. Then, the following equation was used for measuring LRWC:

$$\mathrm{LRWC}\;(\%)=\frac{\text{Fresh weight of leaf}-\text{Dry weight of leaf}}{\text{Turgid weight of leaf}-\text{Dry weight of leaf}}\times 100$$

#### 2.6.3. Estimation of Leaf Malondialdehyde (MDA) Content

Content of MDA was measured using the method given by Cakmak and Horst [58]. Trichloroacetic acid (TCA) method was used for the estimation of MDA content. One g leaf material freshly taken was ground in TCA (10% solution) using a pestle and mortar. The supernatant (0.5 mL) obtained from the homogenized material after centrifugation was mixed with 3 mL of thiobarbituric acid (TBA) prepared in 20% TCA. Test tubes having the solution were kept at 95 °Ċ in a water bath for 50 min, and then cooled immediately. After centrifugation of the resultant mixture at 10,000× *g* for 10 min, the absorbance of colored part was read at 600 and 532 nm. The content of MDA was calculated using the formula:

MDA (nmol) = Δ (A 532 nm − A 600 nm)/1.56 × 105

Absorption coefficient for the calculation of MDA is 156 mmol^−1^ cm^−1^.

#### 2.6.4. Estimation of Leaf H_2_O_2_ Content

Leaf H_2_O_2_ content was determined following by Velikova et al. [59]. The fresh leaf plant samples were ground well in TCA (6%). To 0.1 mL of centrifuged TCA extract added 1 mL of KI solution. The absorbance of resultant mixture was read at 390 nm.

#### 2.6.5. Estimation of Antioxidative Enzyme Activities

*Extraction of Enzymes*

For the extraction of antioxidant enzymes, 0.5 g fresh leaf material was ground in chilled 10 mL of 50 mM phosphate buffer (pH 7.8) using a pestle and mortar. The mixture was then centrifuged at 10,000× *g* for 20 min at 4 °C. The supernatant obtained was then used for the estimation of antioxidative enzymes activities.

*Activity of Superoxide Dismutase (SOD)*

The method ascribed by Giannopolitis and Ries [60] was employed for the estimation of SOD activity measured based on the principle of photochemical reduction inhibition of nitroblue tetrazolium (NBT) in light. The reduction inhibition of NBT was measured at 560 nm. Briefly, the mixture prepared for the study consisted of 1.3 μM riboflavin, 13 mM methionine, 50 μM NBT, 75 nM EDTA, 50 μL enzymatic extract, and 50 mM phosphate buffer (pH 7.8). The mixture was placed in a box coated internally with aluminum foil under a light source of fluorescent light (20 W). The mixture was placed for 15 min under a fluorescent light for the maximum reaction. The absorbance of the reaction mixture was then read at 560 nm. Along with reaction mixture prepared with sample, a reaction mixture without sample was used as a blank. The SOD activity in samples was estimated as unit mg^−1^ protein.

*Determination of Peroxidase (POD), Catalase (CAT), and Ascorbate Peroxidase (APX) Activity*

The method ascribed by Chance and Maehly [61] was followed to assay POD and CAT activities in the leaf buffer extract. The basic mechanism behind the estimation of POD activity was based on the oxidation of guaiacol used in reaction mixture. The decrease in the absorbance of the mixture was taken as a disappearance of H_2_O_2_ as a basic phenomenon behind the estimation of CAT activity. The reaction mixture comprised 1.9 mL H_2_O_2_, 1 mL dH_2_O, and 100 µL of extract. The APX activity was estimated using the method of Asada and Takahashi [62]. The activity of APX was estimated by reading the decrease in the absorbance at 290 nm. The final activities of these enzymes were measured as units mg^−1^ protein.

#### 2.6.6. Estimation of Leaf Ascorbic Acid (AsA) and Tocopherol (Toc) Content

The method ascribed by Mukherjee and Choudhuri [34] was followed for the estimation of AsA content. The standard curve prepared from a range of standard solutions (50–300 ppm) prepared from pure AsA was used to quantify AsA content in the samples. However, for the estimation of leaf total Toc content, the method ascribed by Backer et al. [35] was followed with slight modifications. The extraction of tocopherols from fresh maize leaves was performed using mixture (20 mL for 1 g leaf material) of petroleum ether and ethanol (2:1.6, v/v) using mortar and pastel. The content of the Toc in leaf was calculated using a standard curve prepared with known concentrations of alpha Toc.

#### 2.6.7. Estimation of Leaf Total Flavonoid Content (TFC) and Total Phenolic Content (TPC)

The leaf TFC were estimated following the method as ascribed by Kalita et al. [63]. For the extraction of flavonoids, fresh leaf material (0.5 g) was homogenized in methanol. However, for the estimation of TPC in fresh leaves, the method ascribed by Julkenen-Titto [64] was followed. After homogenization of fresh leaf material (0.05 g) in 5 mL acetone (80%), the homogenate was used for the estimation of TPC. The quantitative estimation of TFC and TPC was done using the standard curves prepared from the known standards.

#### 2.6.8. Estimation of Yield Attributes

At maturity, different yield attributes such as 100 grain weight (100 GW) and grain yield per plant (GY/plant) were estimated manually after harvesting the plants at maturity.

#### 2.6.9. Estimation of Minerals in Root and Shoot

Powdered dry samples of root and shoot were digested using sulfuric acid as ascribed by Wolf [65]. Contents of different cations, such as Ca^+^, K^+^, Mg^2+^, and Fe^2+^ were determined using atomic absorption spectrophotometer (Hitachi. Model 7JO-8024, Tokyo, Japan). Phosphorus content was determined spectrophotometrically using Barton’s reagent. The content of (N) was determined from the digested material using the method ascribed by Bremner and Keeney [66].

#### 2.6.10. Statistical Analysis

Data recorded for varying studied parameters was analyzed statistically using Co-STAT window version 6.3 (developed by Cohort Software Berkley, California, USA) to find out significant differences among the treatments. To find out the significant differences among mean values of different treatments, the least significance difference (LSD) test was employed at 5% level of significance. Correlations and principal component analysis (PCA) of the studied parameters were performed using the XLSTAT software and the significance among the generated values of each attribute was determined using the Spearman’s correlation table.

## 3. Results

Data given in Table 2 shows that PEG-induced water stress significantly affected the G%, CUE, GE, and germination index (GI) of wheat seeds. Seed priming with CRE significantly affected these seed germination attributes under water-stressed and non-stressed conditions, but the increasing or decreasing effects were extract dose-dependent. A significant improvement in these seed attributes was recorded in seeds treated with 10, 20, and 30% CRE and the maximum improvement was at 20% dose of CRE. Similar improvements were also recorded in seeds grown under non-stressed conditions. However, at 40 and 50% levels of CRE, seed priming adversely affected these seed germination attributes and the maximum decrease was at higher level (50%) of the extract (Table 2).

Seed E_50_ and MET were also adversely affected and significantly increased due to PEG-induced water stress. Seed priming with different levels of CRE significantly affected E_50_ and MET of wheat seeds. An improvement was found at lower levels (10, 20, and 30%) of the extract and a maximum decrease was recorded due to seed priming with 20% CRE followed by that recorded at 30% and 10% levels of CRE. Similar improvement in MET and E_50_ was also found under non-stressed conditions. However, the higher levels of CRE (40 and 50%) negatively affected these seed germination attributes under water-stressed and non-stressed conditions, and the maximum adverse effect was recorded at 50% level of CRE (Table 2).

**Figure 1:**
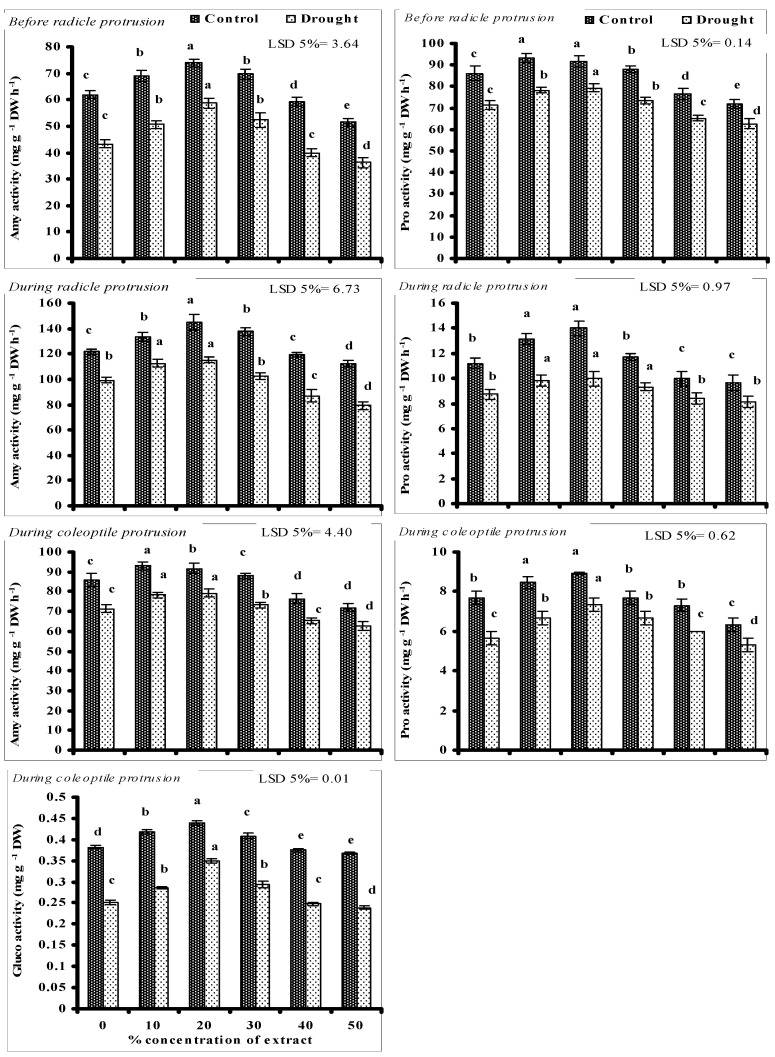
Activities of different germination enzymes of wheat seeds primed with different concentrations of CRE when grown under PEG-induced water stress and non-stressed conditions (mean ± SE; *n*= 3). Similar color bars with same alphabets do not differ significantly.

Data presented in Figure 1 show the activities of seed metabolic enzymes playing a key role in seed germination. Amy and Pro decreased significantly at different seed germination stages of wheat plants grown under PEG-induced water stress without seed priming. Seed priming with lower levels (10, 20, and 30%) of CRE significantly improved the activities of Amy and Pro at all stages of germination and the maximum increase was recorded at 10 and 20% level of CRE under stressed and non-stressed conditions. However, seed priming with higher doses (40 and 50%) of CRE negatively impacted the activities of Amy and Pro at all germination stages under stressed and non-stressed conditions and the maximum decrease was recorded at the 50% level of CRE.

Like Amy and Pro, seed Gluco activity was also decreased significantly when grown under PEG-induced water stress without seed priming. A significant increase in seed Gluco activity was recorded due to seed priming with lower levels of CRE (10, 20, and 30%) under stressed and non-stressed conditions, but the negative effect was recorded at 40 and 50% levels of CRE. Maximum improvement in seed Gluco activity was found due to priming with 20% level of CRE under non-stressed and PEG-induced water stress (Figure 1).

Different morphological and growth attributes, such as root length (RL), shoot fresh weight (SFW), root fresh weight (RFW), shoot dry weight (SDW), and root dry weight (RDW) significantly decreased in water-stressed wheat plants (Table 3). Seed priming with lower doses of CRE found effective in ameliorating the adverse impacts of water stress on these growth attributes and the maximum amelioration due to seed priming was recorded at 10 and 20% levels of CRE. Such improvements in growth attributes due to seed priming with lower doses of CRE were also recorded under non-stressed conditions. However, seed priming with higher doses (40 and 50%) of CRE negatively affected the studied growth attributes under stressed and non-stressed conditions and the maximum decrease was recorded in plants grown from seeds primed with 50% level of CRE.

Significant reductions in different studied yield attributes such as 100 GW and GY/plant were recorded in wheat plants due to imposition of water stress. Seed priming with CRE found effective in reducing the adverse impacts of water stress on these studied yield attributes but this ameliorative effect was recorded only at lower levels of CRE. Seed priming-induced improvement in yield attributes at lower levels of CRE was also recorded under non-stressed conditions. However, the opposite was recorded at higher levels (40 and 50%) of CRE, where a significant reduction was recorded in studied yield attributes under stressed and non-stressed conditions. Seed priming-induced maximum improvement in yield attributes was recorded at 20% level of CRE followed by that recorded at 10 and 30% CRE levels, respectively, while the maximum reduction in yield attributes due to seed priming was recorded at 50% level of CRE (Table 3).

Data presented in Table 4 shows that significant reductions in leaf Chl. *a*, *b*, and total Chl. (T. Chl.) were recorded in water-stressed wheat plants grown without seed priming. Seed priming with CRE was effective in ameliorating the adverse effects of water stress on leaf Chl. *a*, *b*, and T. Chl. However, this ameliorative effect was only recorded at lower doses of CRE (10, 20, and 30%). On the other hand, the higher doses of CRE as seed priming further reduced the leaf Chl. *a*, *b*, and T. Chl. contents. The maximum improvement in leaf Chl. *a*, *b*, and T. Chl. was recorded in plants grown from seeds treated with 20% CRE extract and grown under non-stressed and water-stressed conditions. However, maximum reduction in Chl. *a*, *b*, and T. Chl. was found in plants grown from seeds treated with 50% CRE under non-stressed and water-stressed conditions.

Chl. *a*/*b* significantly increased in water-stressed wheat plants without seed priming. A significant decrease in leaf Chl. *a*/*b* was recorded in plants grown from seeds primed with CRE under stressed and non-stressed conditions. This decrease in Chl. *a*/*b* was CRE dose-dependent and was not the same under stressed and non-stressed conditions. Under water deficit conditions, the decrease in Chl. *a*/*b* was at the maximal level in wheat plants grown from seeds primed with 30% level of CRE. However, under non-stressed conditions, this decrease in Chl. *a*/*b* was higher in wheat plants grown from seeds treated with 20% level of CRE (Table 4). The decrease in Chl. *a*/*b* shows that the increase in Chl. *b* was higher than Chl. *a* due to seed priming with CRE.

Leaf carotenoid (Car.) content of wheat plants increased significantly when grown under water deficit conditions (Table 4). Seed priming with CRE further improved the leaf Car. content of wheat plants grown under water deficit conditions. However, seed priming-induced increase in leaf Car. was found in wheat plants grown from seeds treated with 10, 20, and 30% levels of CRE and the maximum improvement was at 10 and 20% levels of CRE. Seed priming-induced increase in leaf Car. was also found in non-stressed wheat plants.

Imposition of water stress significantly decreased the LRWC of wheat plants when grown without seed priming. Seed priming with lower levels of CRE found effective in maintaining the LRWC of wheat plants when grown under water deficit conditions and the maximum amelioration was found in plants grown from seeds primed with 20% level of CRE. However, seed priming at higher levels (40 and 50%) of CRE caused a further reduction in the LRWC of water stressed wheat plants (Table 4).

Accumulation of MDA and H_2_O_2_ significantly increased in wheat plants grown under water-stressed conditions without seed priming, resulting in increased oxidative damages due to ROS. Significant reduction in leaf MDA and H_2_O_2_ accumulation was recorded in water-stressed wheat plants due to seed priming with CRE but this amelioration was only recorded at 10, 20 and 30% levels of CRE. Comparatively, a maximum reduction was recorded in plants grown from seeds treated with 20% level of CRE. However, at higher levels of CRE, a further increase in H_2_O_2_ and MDA was recorded in water-stressed wheat plants (Table 4).

Significant reductions in K, Ca, N, Mg, and Fe contents in plant shoots and roots were recorded in water-stressed wheat plants grown without seed priming. A significant increase in the nutrients content was recorded in the shoots and roots of wheat plants grown from seeds treated with CRE under stressed and non-stressed conditions. However, seed priming with the lower levels of CRE was found effective in increasing the contents of these nutrients. Additionally, seed priming with higher levels of CRE (40 and 50%) further decreased the contents of these nutrients in roots and shoots of wheat plants grown under stressed and non-stressed conditions (Table 5).

**Figure 2:**
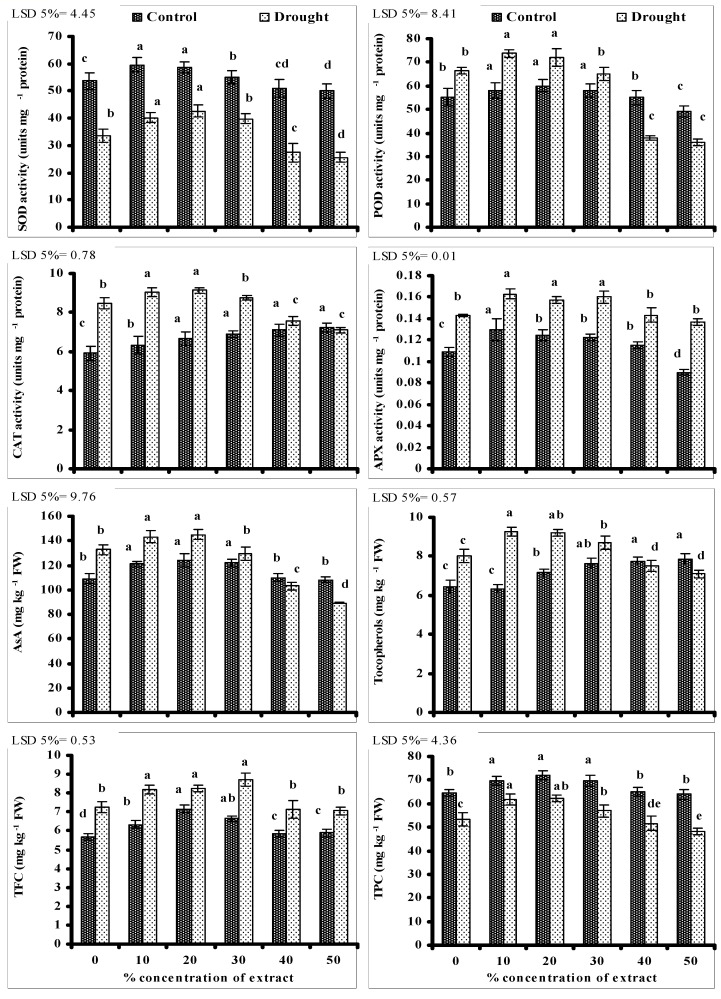
Activities of antioxidative enzymes and levels of non-enzymatic antioxidative compounds of wheat plants grown from seeds primed with different levels of CRE when grown under water-stressed and non-stressed conditions (mean ± SE; *n* = 3). Similar color bars with same letters do not differ significantly.

Data presented in Figure 2 show that SOD activity reduced significantly in water-stressed wheat plants without seed priming. A significant improvement in SOD activity was recorded in water-stressed wheat plants grown from seeds primed with lower levels of CRE. A similar increase in leaf SOD activity due to seed priming with lower levels of CRE was also recorded in the non-stressed wheat plants. The maximum increase in SOD activity was recorded in wheat plants grown from seeds treated with 20% level of CRE. However, a significant decrease in leaf SOD activity was recorded in wheat plants grown from seeds treated with 40 and 50% levels of CRE under stressed and non-stressed conditions (Figure 2).

Leaf POD, CAT, and APX activities increased significantly in wheat plants grown under water stress. Seed priming with different levels of CRE significantly affected the activities of POD, CAT and APX in wheat plants under stressed and non-stressed conditions, but the affect was extract dose-specific. An increase was recorded in wheat plants grown from seeds primed with lower levels of CRE under stressed and non-stressed conditions, but a decrease in the activities of these enzymes was found due to seed priming with 40 and 50% levels of CRE, except to that of leaf CAT activity that increased with increasing CRE levels under non-stressed conditions and the maximum increase was found in wheat plants primed with 40 and 50% levels of CRE.

Imposition of water stress significantly increased the leaf AsA, Toc and flavonoid contents of wheat plants. Seed priming with lower levels of CRE further significantly increased the contents of leaf AsA, Toc and flavonoids of wheat plants but the affect was extract dose-specific. An increase was found in wheat plants grown from seeds primed with lower levels of CRE but a decrease in the contents of these antioxidant compounds was found in plants grown from seeds primed with 40 and 50% levels of CRE. However, leaf Toc content, under non-stressed conditions, increased with increasing CRE levels and a maximum increase was recorded in plants grown from seeds primed with 40 and 50% levels of CRE.

Data presented in Figure 2 show that leaf TPC was significantly reduced in water-stressed wheat plants without seed priming. A significant improvement in leaf TPC was recorded in wheat plants grown from seeds primed with lower levels of CRE. The maximum increase was due to seed priming with 20% level of CRE. A similar increase in leaf TPC was also recorded in the non-stressed wheat plants due to seed priming with lower levels CRE. However, a significant decrease in TPC was found in wheat plants grown from seeds treated with 40 and 50% levels of CRE in comparison with non-treated ones. This decrease in leaf TPC due to 40 and 50% levels of CRE was recorded under stressed and non-stressed conditions (Figure 2).

**Figure 3:**
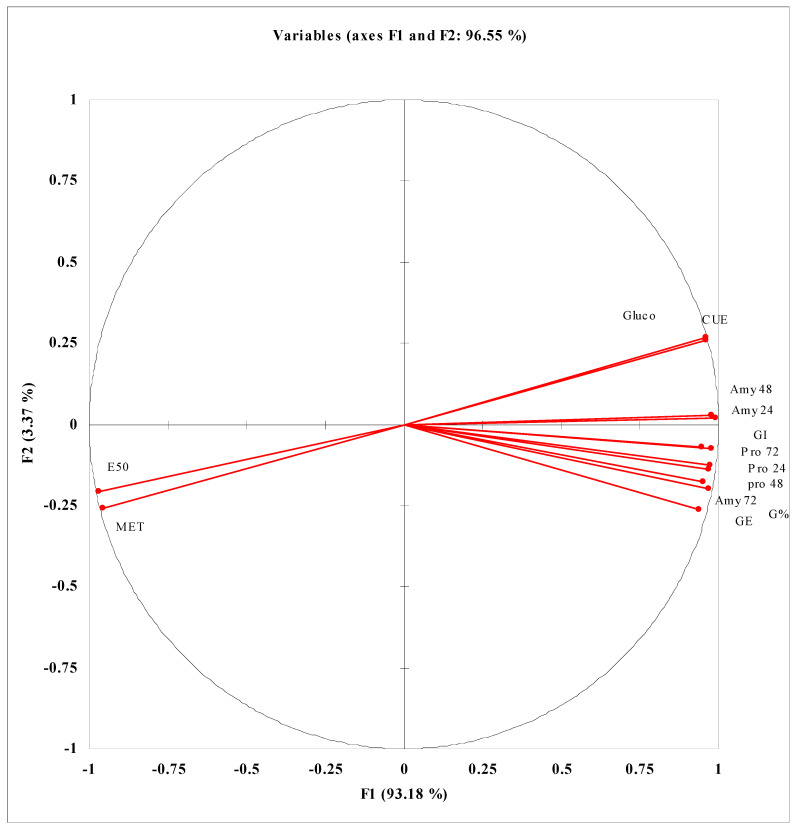
PCA analysis of germination enzyme activities and different germination vigor attributes of wheat seeds treated with different levels of CRE when grown under PEG-induced water stress. Amy I = amylase activity before radicle protrusion; Amy II = amylase activity during radicle protrusion; Amy III = amylase activity during coleoptile protrusion; Pro I = protease activity before radicle protrusion; Pro II = protease activity during radicle protrusion; Pro III = protease activity during coleoptile protrusion; Gluco = glucosidase activity; G% = germination percentage; E_50_ = time to 50% emergence; MET = mean emergence time; CUE = coefficient of uniformity of emergence; GE = germination energy; GI = germination index.

**Figure 4:**
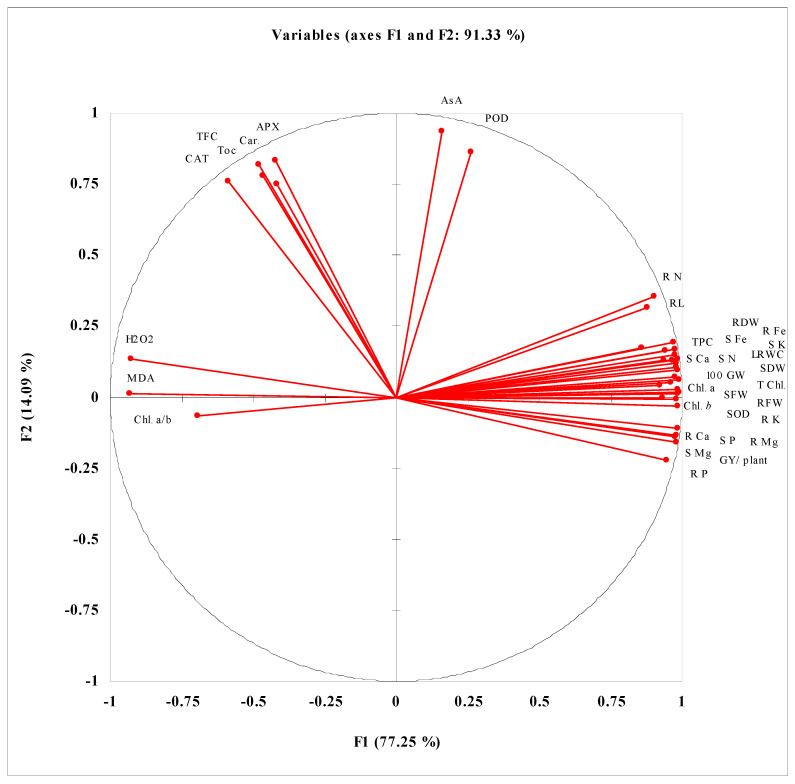
PCA analysis of different growth, yield, physio-biochemical, and nutrient uptake of water-stressed wheat grown from seeds treated with different levels of CRE. SL = shoot length; RL = root length; SFW = shoot fresh weight; RFW-root fresh weight; SDW = shoot dry weight; RDW = root dry weight; NOT = number of tillers; SPKT/SPK = spikelets per spike; 100 GW = hundred grain weight; GY/ plant = grain yield per plant; LRWC = leaf relative water content; Chl. *a* = chlorophyll a; Chl. *b* = chlorophyll b; Chl. *a*/*b* = chlorophyll *a*/*b*; T. Chl. = total chlorophyll; Car. = carotenoids; SOD = superoxide dismutase; POD = peroxidase; CAT = catalase; APX = ascorbate peroxidase; TFC = Total flavonoid content; AsA = ascorbic acid; TPC = Total phenolic content; Toc = tocopherol; MDA = malondialdehyde; S K = shoot K; R K = root K; S Ca = shoot Ca; R Ca = root Ca; S P = shoot P; R P = root P; S N = shoot N; R N = root N; S Mg = shoot Mg; R Mg = root Mg; S Fe = shoot Fe; R Fe = root Fe.

PCA and correlation data presented in Figure 3 and Table S1 show that different studied seed germination attributes such as G%, GE, GI, and CUE have strong positive correlations with activities of seed germination enzyme and are negatively correlated with MET and E_50_. A strong positive correlation of Amy activity with Gluco activity during the germination process at different germination stages has been reported. Among different extracted components, the maximum contribution in determining the variance among the studied attributes is of F1 (93.18) and F2 (3.37) with a cumulative variance of 96.55. Data presented in Table S2 and Figure 4 reveal the correlations of different growth and yield attributes of wheat plants with different physio-biochemical attributes. GY/plant is positively correlated with biomass production, while the biomass production has a strong positive correlation with LRWC, Chl. *a*, Chl. *b*, SOD activity and different nutrients but negatively correlated with MDA and H_2_O_2_. GY/plant is also negatively correlated with leaf MDA and H_2_O_2_ contents. Among different extracted components determining the significant variance among different studied attributes, the maximum contribution is of F1 (77.25) followed by F2 (14.09) with cumulative contribution (91.33) of both factors.

## 4. Discussion

In field crops, under limited irrigation/rainfed conditions, an enhanced crop production is associated with a firm crop stand establishment [67,68]. In this regard, better and fast seed germination is considered important because it contributes significantly in the early establishment of seedlings, especially under soil water deficit conditions [11,69]. Better and fast seed germination is dependent on the activities of seed germination enzymes [11]. In the present study, PEG-induced water stress adversely affected the seed germination-related attributes such as G%, GI, E50, CUE, MET, and GE of wheat seeds. The negative impact of water stress on seed germination attributes to the fact of less absorption of water by wheat seeds due to the physiological shortage of available water in PEG-induced osmotic stress and the reduced activities of germination enzymes [9] that adversely impacted the different germination attributes and seedling emergence. Seed priming with CRE found effective in reducing the adverse impacts of PEG-induced osmotic stress on the studied seed germination attributes and seedling emergence only at the lower doses (10, 20, and 30) and comparatively the maximum improvement was recorded at 20 and 10% level of CRE extract. However, the higher doses of CRE, i.e., 40 and 50% negatively impacted all the studied seed germination-related attributes and the maximum adverse effect was recorded at the 50% level of the extract. This adverse impact of higher doses of CRE was not only found under PEG-induced water stress but also under non-stressed conditions. The improvement in germination-related attributes due to seed priming with CRE might be due to the absorption of nutrients-rich extract by wheat seeds that resulted in lowering the seed internal osmotic potential [11,70] that favored more absorption of water from growth medium. In the present study, CRE was found to be a rich source of different secondary metabolites (flavonoids, phenolics, ascorbic acid), osmolytes, and macro- and micro-mineral nutrients. This reduction in seed osmotic potential resulted in more absorption of water by seeds that boosted the activities of seed germination enzymes, which improved the seed germination potential, seedling emergence and early seedling establishment. Noman et al. [11] reported that wheat seed treatment with sugar beet extract (rich source of osmolytes) significantly improved the seed germination potential and seedling emergence when grown under PEG-induced water stress. Furthermore, it was found that individually applied organic compounds such as phenolics, ascorbic acid, osmolytes etc. as found in CRE resulted in improving the seed germination potential and seedling emergence [14,20]. A similar pattern was found in the present study where the CRE has been found with high concentrations of phenolics and flavonoids that might have improved the germination and seedling emergence. Furthermore, plethora of literature depicts the role of different plant growth regulators such as gibberellic acid, auxins, and cytokinin in seed germination and emergence under normal and stressful environments. It was reported by Ihl et al. [71] that *Cuscuta reflexa* extract is a rich source of gibberellins, auxins and cytokinins (not identified in present study). Thus, the positive influences of CRE as seed priming on seed germination and emergence might be due to the role of phytohormones that might have increased the seed germination and emergence potential of wheat seeds under PEG-induced water stress. Moreover, the nutrients, such as K, N, P, Ca, etc., also play significant roles in maintaining membrane potential, regulating the enzymes activities controlling metabolic activities and maintaining osmotic potential [72,73] and cell division [74]. Potassium maintains better water uptake across cell membranes [75]. However, the high doses of theses nutrients are toxic and adversely affecting the cellular metabolic activities [76]. In the present study, the positive effects of seed priming with low doses of CRE on different germination attributes might be due to the presence of macro- or micro-nutrients that not only reduced the seed osmotic potential for better water uptake but also affected the activities of germination enzymes.

In the present study, the activities of studied enzymes, such as Amy, Pro, and Gluco increased significantly in wheat seeds treated with lower doses of CRE, and the maximum increase was recorded at 10 and 20% level of CRE, resulting in speedy breakdown of seed reserved food materials into simple molecules that are actively used by the newly developing seedlings as building blocks that are helpful in better seed germination and early and fast seedling establishment [9,77]. Moreover, the breakdown of seed reserves into simple compounds further decreases the seed osmotic potential, resulting in higher absorption of water by seeds under water deficit conditions [9]. These findings can be correlated well with the present investigations where lower doses of CRE enhanced the activities of germination enzymes such as Amy, Pro, and Gluco that resulted in more availability of simple metabolites and lowered the seed osmotic potential. The above facts lead to propose that the seed priming with lower doses CRE will helpful in obtaining better yield of wheat under field water deficit conditions. Moreover, the negative impacts of higher levels of CRE as seed priming on the germination and emergence might be due to the adverse effects of active organic compounds on the activities of seed germination enzymes as recorded in the present study. It has been found that higher levels of secondary metabolites adversely affect the seed germination related attributes [78] that could explain the findings of the present study, where higher doses of CRE negatively impacted seed germination and emergence. Irshad and Cheema [79] reported that allelochemicals can inhibit the seed germination by blocking the hydrolysis of seed reserved nutrients, resulting in inhibited cell division. Similarly, Talukder et al. [80] and Radwan et al. [78] reported that the higher doses of leaf aqueous extracts of *Azadirachta indica* and *Calotropis procera* adversely affected the seed germination and seedling growth of turnip and wheat, respectively. They reported that higher concentrations of flavonoids in the extracts negatively affected the seed germination and seedling emergence that can be correlated with the present findings. Furthermore, in the present study, it was found that *Cuscuta reflexa* is rich in phenolics and flavonoids that might have negatively affected the seed germination process at higher doses by restricting the activities of germination enzymes. Ferreira et al. [81] reported that high concentrations of leaf and root extract of *Conyza sumatrensis* adversely affected the seed germination and seedling emergence of *Bidens pilosa*, and this decrease might be attributed to the high levels of phenolics and flavonoids in the extract. Rizzardi et al. [82] reported that 6 and 8% concentration of extracts from the shoots of canola reduced the germination of *Bidens* sp., while Reik [83] recorded a reduction in the germination percentages of *B. pilosa* treated with the aqueous extracts at levels higher than 30% concentration (v/v). In the present study, the high levels of phenolics and flavonoids in CRE at 40 and 50% might have negatively impacted the seed germination and emergence, resulting in reduced plant growth.

Under water deficit conditions, early and better establishment of seedlings firmly depends on the activities of germination enzymes and translocation of metabolites to the developing seedlings that make them tolerant to the adverse environmental conditions [11] that result in better growth and high seed yield. In the present study, seed treatment with lower levels of CRE significantly improved the growth of water-stressed wheat plants in terms of biomass production that finally improved the yield related attributes, where better biomass production is directly associated with better growth of plants [77]. The lower levels of CRE extract (i.e., 10 and 20%) were more effective for use as seed priming. This better growth of wheat plants under water stress is associated with their enhanced seed germination and seedling emergence that might be due to the effect of better translocation of seed reserves to the seedlings [25]. Furthermore, the enhanced growth and yield of water-stressed wheat plants grown from seeds treated with 10 and 20% CRE are associated with their high increase in root biomass and length as well as with their enhanced maintenance of plant water relations in terms of LRWC. The longer and healthy roots are helpful for better uptake of water and nutrients from deeper soil that are also helpful in enhancing tolerance of plants to stressful environment [77].

The enhanced growth and yield of a plant is also associated with a better plant photosynthetic efficiency that directly depends on the status of leaf photosynthetic pigments for capturing the sunlight for use in the photosynthetic processes [84]. The marked devastation of photosynthetic pigments such as leaf Chl. *a*, *b* and T. Chl. under water deficit conditions was recorded, but the content of leaf carotenoids increased. It might be due to the fact that water stress is responsible for inhibition of photosynthetic pigments [18] and perturbations in the ultra-structure of thylakoid membranes [85], resulting in inequality in light capturing and reduced photosynthesis. In the present study, water-stressed wheat plants grown from seeds primed with lower doses of CRE maintained better levels of photosynthetic pigments. The better maintenance of leaf photosynthetic pigments in wheat plants grown from CRE seeds is also associated with their enhanced growth and yield of water-stressed wheat plants. These findings can be correlated with the studies of du Jardin [20] and Noman et al. [11] where the exogenous application of biostimulants and organic extracts significantly enhanced the biosynthesis of photosynthetic pigments that found to be helpful in ameliorating the adverse effects of low water stress. The ameliorating effects of CRE on leaf photosynthetic pigments could be attributed to the fact that it is a rich source of organic compounds, such as phenolics, flavonoids, and vitamins that have a crucial role in the maintenance of chlorophyll structure, photosynthetic machinery, activities of photosynthetic enzymes, and ultra-structures of thylakoid membranes [20] that are helpful in maintaining the better photosynthetic efficiency under various abiotic stresses. The increase in leaf carotenoids of water-stressed wheat plants shows its key role in the tolerance of plants to abiotic stresses, which further increased due to CRE priming. This might be attributed to its role as an antioxidant in protecting the ultra-structure of photosystem-II from over-produced ROS [86]. Similar findings were also reported by the Noman et al. [11] in water-stressed wheat plants, where seed priming with sugar beet extract significantly improved the accumulation leaf photosynthetic pigments, resulting in enhanced plant growth.

Oxidative stress, caused by the excessive production of ROS, is a common phenomenon under different stresses, which reduce the growth and yield of crop plants [87,88]. The extent of oxidative damages is commonly measured in terms of MDA accumulation, due to membrane lipid peroxidation [89,90]. In the water-stressed wheat plants without seed priming, more accumulation of MDA along with high levels of H_2_O_2_ were negatively correlated with the reduced growth and yield, revealing the negative impacts of oxidative stress. To counteract the adversities of overproduced ROS, plants have evolved well-developed anti-oxidative defense mechanisms, including the non-enzymatic ones in terms of secondary metabolites (phenolics, AsA, tocopherols, and flavonoids) that have the ability to capture the ROS and the enzymatic ones that include the activities of CAT, POD, APX, and SOD, which scavenge the ROS [91,92,93]. These both ROS scavenging system work well together in a systematic way [15,18]. In the present study, this ROS scavenging mechanism was not sufficient to counteract the oxidative damages. Seed priming with lower doses of CRE significantly improved the activities of POD, CAT, SOD, and APX as well as the levels of flavonoids, AsA, phenolics, tocopherols, and carotenoids that were negatively correlated with the levels of MDA and H_2_O_2_. It clearly shows that seed priming with lower levels of CRE maintained the better membrane integrity and functioning by boosting the antioxidative defense system, resulting in better growth and yield of water-stressed wheat plants. Furthermore, it has been found that the exogenous application of organic compounds found in the CRE improved the plant stress tolerance [14,15,18].

It is well known that cellular ion homeostasis help in combating the adverse effects of water stress on plant growth. In the present study, significant adverse effects of water stress on the uptake of different nutrient were recorded in the root and shoots of wheat plants, resulting in reduced growth and yield. However, seed priming with lower levels of CRE significantly improved the uptake of these nutrients in the roots and shoot, and the maximum improvement was recorded at 10 and 20% level of CRE. The enhanced uptake of nutrients is the phenomenon of better water uptake from root zone [77,94]. This is also found clearly in the present study where the plants with better water content have better nutrient uptake and is directly related with better root growth.

## 5. Conclusions

This study concludes that CRE can be used as a promising biostimulant. Its lower doses as seed priming found effective in counteracting the adverse effects of water stress on wheat growth and yield. The better growth and seed yield of water-stressed wheat plants grown from seeds treated with lower doses of CRE was associated with their role in improving seed germination and seedling emergence potential due to the enhanced activities of seed germination enzymes. The plants arisen from seeds treated with low doses of CRE tolerated the water stress in a better way, which was associated with their better maintenance of plant water relations, chlorophyll biosynthesis, antioxidative defense mechanism, and better nutrient acquisitions. Future further experimentation is needed to use the best levels of CRE under field water deficit conditions and to find out its impact on seed nutritional quality. Overall, the use of lower doses of CRE is recommended by farmers growing wheat under limited irrigation, especially in rain fed conditions for better yield.

## Figures and Tables

**Table 1:** Biochemical composition of *Cuscuta reflexa* plant extract.

Biochemical/Nutrient	Concentration
Organic	
Total Phenolics	9.08 mg GAE mL^−1^ extract
Total Flavonoids	11.44 mg QE mL^−1^ extract
Ascorbic acid	43.42 µg mL^−1^ extract
Carotenoids	7.26 µg mL^−1^
Tocopherols	20 µg mL^−1^
DPPH activity	IC_50_ 168.6 µg mL^−1^
FRAP (ferric reducing antioxidant power)	40.5 mg GAE mL^−1^
Sugar	5.0% w/v
Protein	11.08% w/v
Moisture	76.0% v/v
Fat	2.4% w/v
Fiber	1.081% w/v
Glycine betaine	1.5 mmol L^−1^ w/v
Proline	0.536 mmol L^−1^ w/v
Inorganic	
K	56 mg mL^−1^
Ca	40 mg mL^−1^
S	0.142 mg mL^−1^
Fe	3 mg mL^−1^
P	3.21 mg mL^−1^
Cl	0.375 mg mL^−1^
Na	2.5 mg mL^−1^
Al	12.3 mg 100 mL^−1^
Zn	2.3 mg 100 mL^−1^
Cu	0.812 mg 100 mL^−1^
Pb	0.45 mg 100 mL^−1^
Co	0.011 mg L^−1^
N	93.6 mg mL^−1^
As	0.0025 mg 100 mL^−1^
Ni	0.356 mg 100 mL^−1^
Mn	1.763 mg 100 m l^−1^
Mg	3.75 mg m L^−1^
Cd	0.0048 mg L^−1^
Cr	0.0453 mg 100 mL^−1^

**Table 2:** Different germination vigor attributes of wheat seeds primed with different levels of *Cuscuta reflexa* extract (CRE) when grown under PEG-induced water stress and non-stressed conditions (mean ± SE; *n* = 4).

	G%		E_50_ (days)		MET (days)		CUE		GE		GI	
Extract Level	Non- Stressed	Water- Stressed	Non- Stressed	Water- Stressed	Non- Stressed	Water- Stressed	Non- Stressed	Water- Stressed	Non- Stressed	Water- Stressed	Non- Stressed	Water- Stressed
0%	90.0 ± 0.58 ^b^	71.70 ± 1.67 ^c^	1.47 ± 0.02 ^ab^	1.86 ± 0.02 ^b^	3.04 ± 0.02 ^c^	3.94 ± 0.02 ^b^	0.38 ± 0.03 ^e^	0.25 ± 0.006 ^c^	0.90 ± 0.006 ^c^	0.75 ± 0.01 ^c^	8.39 ± 0.06 ^d^	6.65 ± 0.09 ^c^
10%	95.3 ± 0.33 ^a^	81.67 ± 2.19 ^b^	1.36 ± 0.01 ^c^	1.69 ± 0.02 ^c^	2.90 ± 0.01 ^d^	3.80 ± 0.05 ^c^	0.42 ± 0.03 ^b^	0.29 ± 0.003 ^b^	0.95 ± 0.006 ^b^	0.80 ± 0.02 ^b^	10.31 ± 0.25 ^b^	7.88 ± 0.09 ^a^
20%	95.3 ± 0.33 ^a^	86.33 ± 2.03 ^a^	1.23 ± 0.02 ^d^	1.61 ± 0.01 ^d^	2.77 ± 0.04 ^e^	3.24 ± 0.02 ^e^	0.44 ± 0.06 ^a^	0.35 ± 0.006 ^a^	1.00 ± 0.000 ^a^	0.85 ± 0.01 ^a^	12.17 ± 0.30 ^a^	7.85 ± 0.02 ^a^
30%	88.3 ± 1.67 ^b^	83.00 ± 0.58 ^ab^	1.33 ± 0.02 ^c^	1.66 ± 0.02 ^e^	2.82 ± 0.02 ^e^	3.65 ± 0.07 ^d^	0.41 ± 0.07 ^b^	0.29 ± 0.009 ^b^	1.00 ± 0.000 ^a^	0.77 ± 0.02 ^c^	9.33 ± 0.33 ^c^	7.25 ± 0.06 ^b^
40%	80.3 ± 2.91 ^c^	70.00 ± 2.31 ^c^	1.46 ± 0.03 ^b^	1.84 ± 0.02 ^b^	3.15 ± 0.02 ^b^	3.99 ± 0.02 ^b^	0.37 ± 0.04 ^c^	0.25 ± 0.004 ^c^	0.77 ± 0.02 ^d^	0.69 ± 0.02 ^d^	8.17 ± 0.17 ^de^	6.76 ± 0.04 ^c^
50%	76.3 ± 0.88 ^d^	67.67 ± 2.03 ^d^	1.52 ± 0.02 ^a^	1.95 ± 0.02 ^a^	3.25 ± 0.04 ^a^	4.10 ± 0.04 ^a^	0.37 ± 0.03 ^c^	0.24 ± 0.003 ^c^	0.71 ± 0.02 ^e^	0.65 ± 0.01 ^d^	7.96 ± 0.08 ^e^	5.96 ± 0.04 ^d^
**LSD 5%**	**3.47**		**0.042**		**0.07**		**0.011**		**0.028**		**0.34**	

Means with same alphabets in superscript in a column do not differ significantly. G% = germination percentage; E_50_ = time to 50% emergence; MET = mean emergence time; CUE = coefficient of uniformity of emergence; GE = germination energy; GI = germination index; LSD = least significance difference.

**Table 3:** Growth and yield attributes of wheat plants grown from seeds primed with different levels of CRE under water-stressed and non-stressed conditions (mean ± SE; *n* = 3).

	**RL (cm)**		**SFW (g plant^−1^)**		**RFW (g plant^−1^)**		**SDW (g plant^−1^)**	
**Extract** **Level**	**Non-** **Stressed**	**Water-** **Stressed**	**Non-** **Stressed**	**Water-** **Stressed**	**Non-** **Stressed**	**Water-** **Stressed**	**Non-** **Stressed**	**Water-** **Stressed**
**0%**	13.90 ± 1.82 ^b^	10.33 ± 0.67 ^b^	33.15 ± 1.15 ^b^	23.41 ± 0.65 ^bc^	8.51 ± 0.35 ^a^	5.41 ± 0.21 ^b^	13.51 ± 0.37 ^b^	8.10 ± 0.35 ^c^
**10%**	16.33 ± 1.17 ^a^	12.83 ± 0.60 ^a^	38.50 ± 0.95 ^a^	28.35 ± 0.75 ^a^	9.45 ± 0.27 ^a^	6.35 ± 0.23 ^a^	15.68 ± 0.25 ^a^	11.25 ± 0.19 ^a^
**20%**	15.33 ± 0.88 ^ab^	13.53 ± 0.79 ^a^	38.51 ± 1.04 ^a^	28.11 ± 0.81 ^a^	9.21 ± 0.21 ^a^	6.75 ± 0.21 ^a^	15.75 ± 0.19 ^a^	10.85 ± 0.18 ^b^
**30%**	13.83 ± 0.60 ^b^	12.00 ± 0.58 ^ab^	33.50 ± 0.85 ^b^	27.11 ± 0.65 ^ab^	8.55 ± 0.34 ^a^	6.45 ± 0.19 ^a^	15.62 ± 0.24 ^a^	10.80 ± 0.21 ^b^
**40%**	11.13 ± 0.68 ^c^	10.10 ± 0.87 ^b^	31.11 ± 0.75 ^bc^	20.32 ± 0.56 ^cd^	6.75 ± 0.27 ^b^	5.10 ± 0.21 ^b^	12.44 ± 0.23 ^b^	8.55 ± 0.18 ^c^
**50%**	11.18 ± 1.30 ^b^	10.07 ± 1.09 ^b^	29.12 ± 0.69 ^c^	18.75 ± 0.45 ^d^	6.35 ± 0.27 ^b^	4.89 ± 0.18 ^b^	10.35 ± 0.17 ^c^	7.21 ± 0.17 ^d^
LSD 5%	2.03		3.15		1.04		1.30	
	**RDW (g plant^−1^)**		**100 GW (g)**		**GY (g plant^−1^)**			
**Extract** **Level**	**Non-** **Stressed**	**Water-** **Stressed**	**Non-** **Stressed**	**Water-** **Stressed**	**Non-** **Stressed**	**Water-** **Stressed**		
**0%**	2.35 ± 0.11 ^b^	1.95 ± 0.11 ^b^	5.33 ± 0.04 ^b^	3.53 ± 0.04 ^b^	13.33 ± 0.20 ^b^	7.33 ± 0.04 ^b^		
**10%**	3.75 ± 0.13 ^a^	2.35 ± 0.10 ^a^	5.76 ± 0.07 ^a^	4.13 ± 0.03 ^a^	14.95 ± 0.01 ^a^	8.36 ± 0.07 ^a^		
**20%**	3.65 ± 0.14 ^a^	2.25 ± 0.09 ^a^	5.81 ± 0.04 ^a^	4.23 ± 0.03 ^a^	15.33 ± 0.02 ^a^	8.60 ± 0.04 ^a^		
**30%**	3.61 ± 0.11 ^a^	2.20 ± 0.11 ^ab^	5.63 ± 0.02 ^a^	3.99 ± 0.04 ^a^	14.90 ± 0.01 ^a^	8.13 ± 0.02 ^b^		
**40%**	2.15 ± 0.10 ^bc^	1.91 ± 0.11 ^c^	4.55 ± 0.04 ^c^	3.01 ± 0.05 ^c^	12.25 ± 0.02 ^c^	6.65 ± 0.04 ^c^		
**50%**	1.95 ± 0.11 ^c^	1.81 ± 0.09 ^c^	4.11 ± 0.03 ^d^	2.76 ± 0.08 ^c^	11.65 ± 0.01 ^d^	5.85 ± 0.03 ^c^		
LSD 5%	0.25		0.35		0.63			

Means with same alphabets in superscript in a column do not differ significantly. RL = root length; SFW = shoot fresh weight; RFW-root fresh weight; SDW = shoot dry weight; RDW = root dry weight; 100 GW = hundred grain weight; GY/plant = grain yield per plant; LSD = least significance difference.

**Table 4:** Leaf photosynthetic pigments, malondialdehyde (MDA), leaf relative water content (LRWC), and H_2_O_2_ levels of wheat plants grown from seeds primed with different levels of CRE under water-stressed and non-stressed conditions (mean ± SE; *n* = 4).

	**Chl. *a* (mg g^−1^ FW)**		**Chl. *b* (mg g^−1^ FW)**		**Chl. *a*/*b***		**T. Chl. (mg g^−1^ FW)**	
**Extract Levels**	**Non-Stressed**	**Water-Stressed**	**Non-Stressed**	**Water-Stressed**	**Non-Stressed**	**Water-Stressed**	**Non-Stressed**	**Water-Stressed**
0%	1.78 ± 0.08 ^ab^	1.48 ± 0.10 ^b^	0.73 ± 0.08 ^bc^	0.55 ± 0.03 ^b^	2.44 ± 0.14 ^b^	2.69 ± 0.28 ^ab^	2.51 ± 0.02 ^bc^	2.03 ± 0.07 ^c^
10%	1.90 ± 0.07 ^ab^	1.68 ± 0.06 ^a^	0.80 ± 0.03 ^ab^	0.66 ± 0.03 ^a^	2.38 ± 0.13 ^bc^	2.55 ± 0.14 ^b^	2.70 ± 0.10 ^ab^	2.34 ± 0.04 ^ab^
20%	1.93 ± 0.11 ^a^	1.81 ± 0.09 ^a^	0.85 ± 0.04 ^a^	0.68 ± 0.10 ^a^	2.27 ± 0.18 ^c^	2.66 ± 0.59 ^ab^	2.78 ± 0.16 ^a^	2.49 ± 0.05 ^a^
30%	1.89 ± 0.08 ^ab^	1.59 ± 0.10 ^b^	0.77 ± 0.02 ^abc^	0.67 ± 0.03 ^ab^	2.45 ± 0.16 ^b^	2.37 ± 0.15 ^c^	2.66 ± 0.12 ^ab^	2.26 ± 0.15 ^b^
40%	1.85 ± 0.15 ^ab^	1.54 ± 0.04 ^b^	0.70 ± 0.05 ^bc^	0.61 ± 0.01 ^ab^	2.64 ± 0.09 ^a^	2.52 ± 0.12 ^bc^	2.56 ± 0.16 ^bc^	2.15 ± 0.08 ^bc^
50%	1.72 ± 0.06 ^b^	1.57 ± 0.07 ^b^	0.69 ± 0.04 ^c^	0.58 ± 0.05 ^ab^	2.49 ± 0.57 ^ab^	2.71 ± 0.23 ^a^	2.41 ± 0.11 ^c^	2.25 ± 0.14 ^b^
**LSD 5%**	**0.28**		**0.10**		**0.15**		**0.21**	
	**Car. (mg g^−1^ FW)**		**LRWC (%)**		**MDA (nmol g^−1^ FW)**		**H_2_ O_2_ (µmol g^−1^ FW)**	
**Extract Levels**	**Non-Stressed**	**Water-Stressed**	**Non-Stressed**	**Water-Stressed**	**Non-Stressed**	**Water-Stressed**	**Non-Stressed**	**Water-Stressed**
0%	0.061 ± 0.64 ^c^	0.080 ± 0.64 ^b^	82 ± 1.41 ^b^	68 ± 1.14 ^b^	23.0 ± 0.58 ^a^	44.7 ± 1.76 ^b^	2.03 ± 0.12 ^a^	5.61 ± 0.29 ^b^
10%	0.072 ± 0.02 ^ab^	0.093 ± 0.02 ^e^	85 ± 1.81 ^a^	74 ± 2.03 ^a^	22.0 ± 1.73 ^a^	34.0 ± 2.65 ^c^	1.99 ± 0.05 ^a^	4.10 ± 0.21 ^c^
20%	0.075 ± 0.02 ^a^	0.094 ± 0.02 ^e^	85 ± 2.04 ^a^	75 ± 1.23 ^a^	23.0 ± 0.58 ^a^	32.8 ± 4.69 ^c^	1.96 ± 0.10 ^a^	4.07 ± 0.19 ^c^
30%	0.071 ± 0.01 ^abc^	0.087 ± 0.04 ^ab^	81 ± 1.02 ^b^	65 ± 1.41 ^c^	24.7 ± 3.18 ^a^	36.1 ± 3.18 ^c^	1.95 ± 0.03 ^a^	3.98 ± 0.18 ^c^
40%	0.065 ± 0.02 ^abc^	0.081 ± 0.05 ^b^	76 ± 1.44 ^c^	63 ± 1.15 ^cd^	25.3 ± 1.76 ^a^	48.4 ± 5.91 ^a^	2.06 ± 0.17 ^a^	5.79 ± 0.19 ^a^
50%	0.062 ± 0.05 ^bc^	0.079 ± 0.05 ^b^	68 ± 1.03 ^d^	62 ± 1.35 ^d^	24.5 ± 0.78 ^a^	49.3 ± 4.09 ^a^	2.08 ± 0.12 ^a^	5.87 ± 0.12 ^a^
**LSD 5%**	**0.01**		**2.61**		**4.10**		**0.15**	

Means with same alphabets in superscript in a column do not differ significantly. Chl. *a* = chlorophyll a; Chl. *b* = chlorophyll b; Chl. *a*/*b* = chlorophyll *a*/*b*; T. Chl. = total chlorophyll; Car. = carotenoids; LRWC = leaf relative water content; MDA = malondialdehyde; LSD = least significance difference.

**Table 5:** Nutrient levels of wheat plants grown from seeds primed with different levels of CRE under water-stressed and non-stressed conditions (mean ± SE; *n* = 3).

	**S K (mg g^−1^ DW)**		**R K (mg g^−1^ DW)**		**S Ca (mg g^−1^ DW)**		**R Ca (mg g^−1^ DW)**		**S P (mg g^−1^ DW)**		**R P (mg g^−1^ DW)**	
**Extract Level**	**Non-** **Stressed**	**Water-** **Stressed**	**Non-** **Stressed**	**Water-** **Stressed**	**Non-** **Stressed**	**Water-** **Stressed**	**Non-** **Stressed**	**Water-** **Stressed**	**Non-** **Stressed**	**Water-** **Stressed**	**Non-** **Stressed**	**Water-** **Stressed**
**0%**	36.30 ± 1.56 ^bc^	27.50 ± 1.44 ^c^	33.65 ± 1.88 ^b^	22.90 ± 1.96 ^b^	7.67 ± o.88 ^b^	5.33 ± 0.29 ^b^	3.46 ± 0.37 ^b^	2.01 ± 0.02 ^b^	2.40 ± 0.05 ^c^	1.57 ± 0.02 ^b^	2.50 ± 0.04 ^c^	1.36 ± 0.02 ^c^
**10%**	40.10 ± 1.67 ^a^	32.50 ± 2.02 ^a^	36.70 ± 2.19 ^a^	27.85 ± 1.53 ^a^	8.34 ± 0.38 ^a^	6.33 ± 0.39 ^a^	3.91 ± 0.12 ^a^	2.52 ± 0.22 ^a^	2.71 ± 0.0 ^a^	1.95 ± 0.04 ^a^	2.71 ± 0.01 ^a^	1.65 ± 0.03 ^a^
**20%**	39.50 ± 0.87 ^ab^	31.60 ± 2.08 ^ab^	37.15 ± 0.66 ^a^	26.34 ± 1.25 ^a^	8.48 ± 0.21 ^a^	6.29 ± 0.27 ^a^	4.07 ± 0.48 ^a^	2.55 ± 0.19 ^a^	2.60 ± 0.03 ^b^	1.91 ± 0.02 ^a^	2.60 ± 0.05 ^b^	1.71 ± 0.01 ^a^
**30%**	41.00 ± 1.16 ^a^	31.00 ± 1.73 ^bc^	37.50 ± 2.02 ^a^	26.03 ± 2.76 ^a^	8.22 ± 0.13 ^a^	6.11 ± 0.16 ^a^	3.90 ± 0.21 ^a^	2.51 ± 0.12 ^a^	2.72 ± 0.01 ^a^	1.87 ± 0.01 ^a^	2.72 ± 0.01 ^a^	1.56 ± 0.02 ^b^
**40%**	33.05 ± 0.78 ^c^	24.00 ± 2.31 ^bc^	30.45 ± 1.76 ^c^	20.70 ± 0.81 ^bc^	6.74 ± 0.14 ^c^	5.14 ± 0.42 ^b^	3.32 ± 0.15 ^b^	1.97 ± 0.15 ^b^	2.31 ± 0.04 ^cd^	1.60 ± 0.12 ^b^	2.51 ± 0.02 ^c^	1.30 ± 0.01 ^c^
**50%**	32.40 ± 1.21 ^c^	23.25 ± 1.31 ^c^	29.40 ± 0.35 ^c^	19.90 ± 2.78 ^c^	6.27 ± 0.35 ^c^	4.90 ± 0.36 ^b^	3.27 ± 0.10 ^b^	1.85 ± 0.10 ^b^	2.14 ± 0.03 ^d^	1.40 ± 0.09 ^c^	2.44 ± 0.18 ^c^	1.20 ± 0.00 ^d^
LSD 5%	3.26		2.90		0.78		0.25		0.10		0.07	
	**S N (mg g^−1^ DW)**		**R N (mg g^−1^ DW)**		**S Mg (mg g^−1^ DW)**		**R Mg (mg g^−1^ DW)**		**S Fe (mg 100 g^−1^ DW)**		**R Fe (mg 100 g^−1^ DW)**	
**Extract Level**	**Non-** **Stressed**	**Water-** **Stressed**	**Non-** **Stressed**	**Water-** **Stressed**	**Non-** **Stressed**	**Water-** **Stressed**	**Non-** **Stressed**	**Water-** **Stressed**	**Non-** **Stressed**	**Water-** **Stressed**	**Non-** **Stressed**	**Water-** **Stressed**
**0%**	45.30 ± 1.56 ^c^	29.50 ± 1.44 ^c^	33.65 ± 1.88 ^bc^	25.90 ± 1.96 ^c^	3.13 ± 0.17 ^b^	1.71 ± 0.09 ^b^	2.37 ± 0.17 ^b^	1.13 ± 0.09 ^bc^	32.00 ± 0.85 ^d^	19.00 ± 0.35 ^d^	26.25 ± 0.38 ^c^	14.50 ± 0.25 ^c^
**10%**	48.10 ± 1.67 ^ab^	37.50 ± 2.02 ^a^	37.66 ± 2.19 ^a^	33.95 ± 1.53 ^a^	3.35 ± 0.33 ^a^	1.94 ± 0.05 ^a^	2.92 ± 0.08 ^a^	1.48 ± 0.07 ^a^	42.50 ± 1.35 ^a^	25.43 ± 0.47 b^b^	33.00 ± 0.58 ^a^	20.00 ± 0.33 ^a^
**20%**	49.50 ± 0.87 ^a^	38.60 ± 2.08 ^a^	37.15 ± 0.66 ^a^	34.34 ± 1.25 ^a^	3.41 ± 0.17 ^a^	1.98 ± 0.07 ^a^	2.97 ± 0.17 ^a^	1.44 ± 0.05 ^a^	39.17 ± 0.95 ^b^	28.00 ± 0.53 ^a^	31.17 ± 0.78 ^ab^	21.50 ± 0.35 ^a^
**30%**	46.00 ± 1.16 ^b^	35.00 ± 1.73 ^b^	35.50 ± 2.02 ^b^	31.03 ± 2.76 ^b^	3.29 ± 0.08 ^a^	1.72 ± 0.07 ^b^	2.47 ± 0.33 ^b^	1.30 ± 0.03 ^ab^	35.00 ± 0.63 ^c^	22.00 ± 0.27 ^c^	29.25 ± 0.53 ^b^	18.43 ± 0.58 ^b^
**40%**	37.95 ± 0.78 ^d^	27.00 ± 2.31 ^d^	32.45 ± 1.76 ^c^	25.10 ± 0.81 ^d^	2.42 ± 0.15 ^c^	1.60 ± 0.03 ^bc^	2.02 ± 0.15 ^c^	1.08 ± 0.03 ^bc^	24.00 ± 1.01 ^e^	18.00 ± 0.49 ^d^	19.73 ± 0.45 ^d^	11.50 ± 0.41 ^d^
**50%**	34.40 ± 1.21 ^e^	24.25 ± 1.31 ^e^	32.40 ± 0.35 ^c^	21.99 ± 2.78 ^d^	2.37 ± 0.17 ^c^	1.47 ± 0.03 ^c^	1.95 ± 0.17 ^c^	1.02 ± 0.07 ^c^	21.83 ± 0.98 ^e^	17.00 ± 0.37 ^d^	18.83 ± 0.35 ^d^	10.50 ± 0.28 ^d^
LSD 5%	2.25		2.10		0.21		0.25		**2.50**		**2.15**	

Means with same alphabets in superscript in a column do not differ significantly. S K = shoot K; R K = root K; S Ca = shoot Ca; R Ca = root Ca; S P = shoot P; R P = root P; S N = shoot N; R N = root N; S Mg = shoot Mg; R Mg = root Mg; S Fe = shoot Fe; R Fe = root Fe; LSD = least significance difference.

## Supplementary Materials

The following are available online at https://www.mdpi.com/2218-273X/10/9/1212/s1, Table S1: Pearson’s correlation coefficient values of seed germination enzymes and germination vigor attributes, Table S2: Pearson’s correlation coefficient values of different growth, yield, physio-biochemical attributes and nutrient content of water-stressed wheat plants grown from seeds treated with different levels of CRE.
