# Supplementary material for: Low Doses of Cuscuta reflexa Extract Act as Natural Biostimulants to Improve the Germination Vigor, Growth, and Grain Yield of Wheat Grown under Water Stress: Photosynthetic Pigments, Antioxidative Defense Mechanisms, and Nutrient Acquisition
Source: Biomolecules. 2020 Aug 20;10(9):1212. doi: 10.3390/biom10091212 (PMC7565113; doi:10.3390/biom10091212)
Supplement: Supplementary file 1 [file biomolecules-10-01212-s001.pdf]

Table S1. Pearson's correlation coefficient values of seed germination enzymes and germination vigor attributes.

Variables	Amy I	Amy II	Amy III	Pro I	Pro II	Pro III	Gluco
**Amy I**	**1**	0.980***	0.957***	0.958***	0.938***	0.970***	0.956***
**Amy II**	0.980***	**1**	0.952***	0.943***	0.931***	0.934***	0.941***
**Amy III**	0.957***	0.952***	**1**	0.964***	0.954***	0.947***	0.881***
**Pro I**	0.958***	0.943***	0.964***	**1**	0.976***	0.950***	0.905***
**Pro II**	0.938***	0.931***	0.954***	0.976***	**1**	0.957***	0.899***
**Pro III**	0.970***	0.934***	0.947***	0.950***	0.957***	**1**	0.918***
**Gluco**	0.956***	0.941***	0.881***	0.905***	0.899***	0.918***	**1**
**G%**	0.950***	0.910***	0.966***	0.917***	0.925***	0.964***	0.868***
**E_{50}**	−0.970***	−0.964***	−0.892***	−0.920***	−0.909***	−0.931***	−0.982***
**MET**	−0.962***	−0.940***	−0.883***	−0.902***	−0.877***	−0.911***	−0.993***
**CUE**	0.954***	0.944***	0.884***	0.907***	0.903***	0.917***	0.999***
**GE**	0.939***	0.926***	0.966***	0.957***	0.920***	0.911***	0.831***
**GI**	0.916***	0.925***	0.897***	0.939***	0.978***	0.938***	0.887***

*** = significant at 0.001 level

Amy I = amylase activity before radicle protrusion; Amy II = amylase activity during radicle protrusion; Amy III = amylase activity during coleoptile protrusion; Pro I = protease activity before radicle protrusion; Pro II = protease activity during radicle protrusion; Pro III = protease activity during coleoptile protrusion; Gluco = glucosidase activity; G% = germination percentage; E_{50} = time to 50% emergence; MET = mean emergence time; CUE = coefficient of uniformity of emergence; GE = germination energy; GI = germination index

Table S2. Pearson's correlation coefficient values of different growth, yield, physio-biochemical attributes and nutrient content of water-stressed wheat plants grown from seeds treated with different levels of CRE.

Variables	RL	SFW	RFW	SDW	RDW	100 GW	GY/plant
**RL**	**1**	0.895***	0.938***	0.890***	0.875***	0.897***	0.778***
**SFW**	0.895***	**1**	0.963***	0.960***	0.839***	0.977***	0.944***
**RFW**	0.938***	0.963***	**1**	0.972***	0.892***	0.985***	0.934***
**SDW**	0.890***	0.960***	0.972***	**1**	0.904***	0.982***	0.943***
**RDW**	0.875***	0.839***	0.892***	0.904***	**1**	0.870***	0.820***
**100 GW**	0.897***	0.977***	0.985***	0.982***	0.870***	**1**	0.956***
**GY/ plant**	0.778***	0.944***	0.934***	0.943***	0.820***	0.956***	**1**
**LRWC**	0.899***	0.935***	0.945***	0.932***	0.831***	0.957***	0.893***
**Chl. *a***	0.809***	0.893***	0.871***	0.905***	0.776***	0.888***	0.891***
**Chl. *b***	0.879***	0.945***	0.945***	0.958***	0.868***	0.934***	0.925***
**Chl. *a*/*b***	−0.675***	−0.703***	−0.731***	−0.722***	−0.686***	-0.688***	−0.662***
**T. Chl.**	0.831***	0.898***	0.894***	0.917***	0.815***	0.893***	0.902***
**Car.**	−0.086ns	−0.392*	−0.384*	−0.362*	−0.173ns	−0.408*	−0.620***
**SOD**	0.813***	0.976***	0.929***	0.932***	0.764***	0.960***	0.969***
**POD**	0.435**	0.389*	0.295ns	0.308ns	0.243ns	0.367*	0.140ns
**CAT**	−0.351*	−0.477**	−0.568***	−0.514**	−0.445**	−0.519**	−0.675***
**APX**	−0.074ns	−0.376*	−0.341*	−0.319ns	−0.143ns	−0.373*	−0.603***
**TFC**	−0.068ns	−0.329ns	−0.325ns	−0.301ns	−0.135ns	−0.344*	−0.557***
**AsA**	0.390*	0.281ns	0.201ns	0.223ns	0.235ns	0.260ns	0.028ns
**TPC**	0.832***	0.966***	0.909***	0.944***	0.803***	0.951***	0.940***
**Toc**	−0.276ns	−0.327ns	−0.448**	−0.350*	-0.372*	−0.365*	-0.498**
**MDA**	−0.748***	−0.932***	−0.863***	−0.876***	−0.646***	−0.906***	−0.922***
**H_2O_2**	−0.684***	−0.905***	−0.842***	−0.870***	−0.638***	−0.887***	−0.939***
**S K**	0.879***	0.965***	0.957***	0.974***	0.872***	0.986***	0.942***
**R K**	0.849***	0.968***	0.962***	0.978***	0.864***	0.986***	0.979***
**S Ca**	0.903***	0.969***	0.988***	0.991***	0.900***	0.994***	0.961***
**R Ca**	0.790***	0.954***	0.932***	0.950***	0.819***	0.954***	0.994***
**S P**	0.823***	0.956***	0.946***	0.977***	0.847***	0.971***	0.982***
**R P**	0.693***	0.905***	0.867***	0.890***	0.712***	0.905***	0.978***
**S N**	0.933***	0.974***	0.980***	0.975***	0.857***	0.990***	0.916***
**R N**	0.882***	0.938***	0.878***	0.903***	0.756***	0.909***	0.829***
**S Mg**	0.832***	0.932***	0.962***	0.947***	0.856***	0.964***	0.982***
**R Mg**	0.840***	0.957***	0.956***	0.944***	0.857***	0.953***	0.982***
**S Fe**	0.973***	0.932***	0.976***	0.946***	0.935***	0.948***	0.877***
**R Fe**	0.954***	0.971***	0.989***	0.972***	0.910***	0.985***	0.921***

***, ** and * = significant at 0.001, 0.01 and 0.05 levels respectively.

RL = root length; SFW = shoot fresh weight; RFW-root fresh weight; SDW = shoot dry weight; RDW = root dry weight; 100 GW = hundred grain weight; GY/ plant = grain yield per plant; LRWC = leaf relative water content; Chl. *a* = chlorophyll a; Chl. *b* = chlorophyll b; Chl. *a*/*b* = chlorophyll *a*/*b*; T. Chl.= total chlorophyll; Car.= carotenoids; SOD = superoxide dismutase; POD = peroxidase; CAT = catalase; APX = ascorbate peroxidase; TFC = Total flavonoid content; AsA = ascorbic acid; TPC = Total phenolic content; Toc = tocopherol; MDA = malondialdehyde; S K = shoot K; R K = root K; S Ca = shoot Ca; R Ca = root Ca; S P = shoot P; R P = root P; S N = shoot N; R N = root N; S Mg = shoot Mg; R Mg = root Mg; S Fe = shoot Fe; R Fe = root Fe.
